# Short and long term determinants of incident multimorbidity in a cohort of 1988 earthquake survivors in Armenia

**DOI:** 10.1186/1475-9276-12-68

**Published:** 2013-08-20

**Authors:** Anahit Demirchyan, Vahe Khachadourian, Haroutune K Armenian, Varduhi Petrosyan

**Affiliations:** 1 School of Public Health, American University of Armenia, 40 Marshal Baghramian Avenue, Yerevan 0019, Armenia; 2 Department of Epidemiology, Fielding School of Public Health, UCLA, Los Angeles, CA, USA

**Keywords:** Multimorbidity, Determinants, Inequity, Life events, Earthquake, Cohort, Armenia

## Abstract

**Background:**

Multimorbidity, presence of two or more health conditions, is a widespread phenomenon affecting populations’ health all over the world. It becomes a serious public health concern due to its negative consequences on quality of life, mortality, and cost of healthcare services utilization. Studies exploring determinants of multimorbidity are limited, particularly those looking at vulnerable populations prospectively over time. This study aimed at identifying short and long term socioeconomic, psychosocial, and health behavioral determinants of incident multimorbidity among a cohort of the 1988 Armenian earthquake survivors.

**Methods:**

The study included a representative subsample of 725 from a larger initial cohort of the earthquake survivors. Data on this subsample were collected via four phases of this cohort study during the period 1990–2012. The final logistic regression analysis eliminated all those cases with baseline multimorbidity to investigate short and long term determinants of incident multimorbidity; this subsample included 600 participants.

**Results:**

More than 75% of the studied sample had multimorbidity. Perceived low affordability of healthcare services, poor living standards during the post-earthquake decade, and lower education were independent predictors of incident multimorbidity developed during the period 1990–2012. Stressful life events and poor social support were among psychosocial determinants of incident multimorbidity. Participants’ baseline BMI reported in 1990 was independently associated with incident multimorbidity.

**Conclusions:**

Most of the identified determinants of incident multimorbidity in our study population were markers of social inequities, indicating that inequities pose a serious threat to both individual and public health-related outcomes. Strategies targeting to decrease such inequities along with promotion of healthy lifestyle and strengthening of social networks may considerably reduce multimorbidity among population groups with similar socioeconomic and cultural profiles.

## Background

Multimorbidity is a condition during which an individual suffers from co-occurrence of two or more health problems [1]. Although there are significant variations in research methodology and operational definition of the condition across studies estimating the prevalence rates of multimorbidity [2-5], the majority of findings consistently indicate that multimorbidity is a common public health problem [3,6-9]. Recent improvements in living standards and advancements in prevention and treatment of communicable diseases have resulted in an increase of life expectancy and consequently in a higher number and proportion of elderly population contributing to higher rates of non-communicable diseases. However, multimorbidity is not a problem limited to elderly population and could be a significant problem among those below the age of 65 [6,10].

Multimorbidity is associated with various adverse consequences and health outcomes. Those with multimorbidity have been found to have significantly higher rates of health services utilization and eventually higher medical expenditure than those without [8,11]. Negative impacts of multimorbidity on the quality of life and functioning have been well established in the literature [9,12-15]. Multimorbidity is also associated with poorer self reported health [16]. Researchers have also found that those with multimorbidity have higher rates of mortality [9,17,18].

Several studies have focused on exploring risk factors and determinants of multimorbidity [6,19-22] and suggested that the risk of multimorbidity increases with age. Inequities are also associated with multimorbidity [23]. Socioeconomic status is consistently found to be an important determinant of multimorbidity [6,19,20,24]. Insufficient nutrition and food intake are significantly associated with higher risks of multimorbidity [25] highlighting the relation between inequities and multimorbidity. Additionally, obesity is also found to be associated with multimorbidity [19].

A majority of studies examining the relation between education and multimorbidity indicated that lower education is an independent determinant of multimorbidity [21,22,26]. Very few studies have also explored the psychosocial determinants of multimorbidity: low levels of social support and living alone were among the factors contributing to multimorbidity [19,27].

Despite the high rates and burden of multimorbidity, recent review papers noted that only a limited number of studies aimed at exploring determinants and risk factors of multimorbidity [9,15]. To better deal with consequences of such an adverse phenomenon, a better understanding of the risk factors for multimorbidity among diverse study populations becomes essential.

In December 1988 an earthquake with a magnitude of 6.9 on the Richter scale struck the northern part of Armenia, damaging one-third of the countries’ territory and making more than 500,000 people homeless. The number of those injured exceeded 100,000 and over 25,000 died. Shortly after the earthquake, in early 1990’s, the very difficult socio-economic transition from Soviet to independent Armenia further affected populations’ living standards. In 1990, a large representative sample of earthquake survivors from the biggest cities in the north of Armenia was involved in a longitudinal study of physical and mental health outcomes [28]. A subsample of this cohort was followed for 22 years, providing a unique opportunity to investigate the long-term effects of certain baseline characteristics of the survivors on their current health outcomes. The purpose of this study was to examine the role of survivors’ selected baseline and current characteristics on newly occurring multimorbidity during 1990–2012. Those characteristics were grouped in socioeconomic, health behavior, and psychosocial dimensions.

## Methods

### Participants

The study participants were a sample of 725 individuals from a cohort of the 1988 earthquake survivors. The initial cohort consisted of all employees of the healthcare services in the earthquake zone and their family members – 32,743 individuals, who participated in a baseline assessment in 1990. A year later, a psychopathological investigation was carried out among 1,785 individuals of this cohort selected through geographically stratified random sampling to achieve higher representation from areas most affected by the earthquake [29,30]. Eighty percent of this study group was followed in 2012 and 725 individuals participated in a complete follow-up assessment. Of these, 124 had multimorbidity at baseline and were eliminated from the final analysis to explore the determinants of incident multimorbidity. The studied sample was representative for the population of the earthquake area aged 40 and over in terms of its major characteristics [31]. The baseline assessments collected detailed data on respondents’ earthquake-related experiences and outcomes, self-reported physical and mental health status, health behavior, access to healthcare services, and socio-demographic characteristics. The 2012 assessment collected data on respondents’ self-reported physical and mental health status, health behavior, access to healthcare services, and socio-demographic characteristics more than two decades after the baseline assessments. The Institutional Review Board of the American University of Armenia reviewed and approved the protocol for the current study.

### Variables

The outcome variable, *incident multimorbidity*, was based on the number of respondent’s self-reported non-communicable health conditions at the current assessment. Respondents were provided with a predetermined list of conditions (including both physical and mental conditions) and the option “other” to make the listing of conditions comprehensive. This variable was dichotomized into two or more diseases versus one or no disease.

The independent variables reflected characteristics collected at two time points – in 1990s and in 2012 follow-up survey, and were grouped into three dimensions: socioeconomic, health behavior, and psychosocial. The socioeconomic dimension included age, gender, education, employment, marital status, living alone, perceived low affordability of healthcare services, socio-economic status (SES) score (ranging from 0 to 24), and perceived living standards before the earthquake and during the first decade after the earthquake. The dimension of health behavior included smoking, drinking alcohol once a week or more, binge drinking, uncontrolled use of salt, body mass index (BMI), regular participation in sport and walking three hours per week or more. The psychosocial dimension included earthquake-caused material loss score (ranging from 0 to 8), earthquake-related deaths in the family, earthquake-related injuries, post-traumatic growth score (ranging from 0 to 20), dignity score (ranging from 18 to 90), number of stressful life events, memory score as an indicator of cognitive functioning (ranging from 0 to 8), and social support. Some of these variables were available either from the baseline or current assessments, some – from both assessments (Table 1).

**Table 1:** Distribution of selected baseline and current variables between groups with and without multimorbidity among a cohort of the 1988 Armenian earthquake survivors

	**n**	**Baseline multi-morbidity (N=124)**	**Incident multimorbidity**			**Total (N=721)**
			**No (N=157)**	**Yes (N=440)**	***P value***	
*Socioeconomic variables:*						
Current age, mean (SD)	721	64.5 (10.4)	53.6 (10.5)	58.4 (12.1)	*0.000*	58.4 (12.0)
Current socio-economic status score, mean (SD)	713	12.0 (3.8)	13.3 (4.2)	12.0 (3.9)	*0.000*	12.3 (4.0)
Gender:						
Female, %	721	81.5	62.4	66.4	*0.381*	68.1
Male, %		18.5	37.6	33.6		31.9
Current education:						
Secondary/lower, %	721	83.1	61.6	80.0	*0.000*	69.1
University/higher, %		16.9	38.9	20.0		23.6
Current employment:						
Employed, %	721	41.1	58.6	41.1	*0.000*	44.9
Unemployed, %		58.9	41.4	58.9		55.1
Current marital status:						
Married, %	721	58.9	72.6	72.3	*1.000*	70.0
Single/divorced/widowed, %		41.1	27.4	27.7		30.0
Currently living alone, %	720	9.8	7.0	6.1	*0.705*	6.9
Current low affordability of healthcare, %	720	61.3	35.7	64.9	*0.000*	57.9
Living standards during post-earthquake decade:						
Very good/good/average, %	717	45.5	67.5	48.3	*0.000*	52.0
Poor/very poor, %		54.5	32.5	51.7		48.0
Baseline living standards:						
Good/average, %	716	60.5	72.7	73.1	*1.000*	70.8
Poor/very poor, %		39.5	27.3	26.9		29.2
*Health behavioral variables*:						
Current smoking, %	721	5.6	24.2	20.0	*0.305*	18.4
Current drinking once a week or more, %	716	4.9	16.0	10.3	*0.061*	10.6
Current binge drinking, %	713	4.9	8.4	7.6	*0.728*	7.3
Current regular walking (≥3 hours/week), %	716	33.3	45.9	36.0	*0.035*	37.7
Current uncontrolled use of salt, %	716	22.1	26.1	31.1	*0.264*	28.5
Baseline smoking, %	721	8.9	24.8	20.2	*0.257*	19.3
Baseline drinking (perceived), %	721	29.0	23.6	25.5	*0.669*	25.7
Baseline regular participation in sport, %	716	2.4	9.0	4.3	*0.041*	5.0
Baseline body mass index, mean (SD)	713	26.2 (4.4)	24.2 (3.9)	25.5 (4.6)	*0.002*	25.3 (4.5)
*Psychosocial variables:*						
Current good social support, %	706	49.2	77.9	54.7	*0.000*	58.8
Current post-traumatic growth score, mean (SD)	694	9.8 (5.0)	10.3 (5.0)	9.6 (5.2)	*0.144*	9.8 (5.1)
Current dignity score, mean (SD)	698	70.8 (7.7)	71.6 (6.9)	70.1 (6.9)	*0.018*	70.6 (7.1)
Current memory score, mean (SD)	719	4.6 (2.2)	5.4 (2.1)	4.7 (2.2)	*0.001*	4.8 (2.2)
Number of stressful life events, mean (SD)	721	9.4 (2.7)	7.5 (2.6)	8.7 (2.7)	*0.000*	8.6 (2.7)
Earthquake-caused material loss score, mean (SD)	716	3.0 (1.9)	2.5 (2.0)	2.6 (2.0)	*0.589*	2.7 (2.0)
Earthquake-related deaths in the family, %	721	10.5	7.6	9.8	*0.521*	9.4
Earthquake-related injuries, %	721	16.9	7.6	6.6	*0.713*	8.6

*N* number of respondents in each group, *n* number of valid responses, *SD* Standard deviation. *P value* two-sided.

Age, BMI, number of stressful life events and all the score variables were treated as continuous variables in logistic regression analysis after checking their linearity on logistic scale. The remaining variables were dichotomized. All the dichotomized variables were single-item variables, except social support. This variable was based on a cumulative score generated from the responses to seven social support items and then dichotomized at a threshold corresponding to its median as this score demonstrated parabolic pattern on the logistic scale.

### Analysis

The main characteristics of the sample were investigated through descriptive analysis and compared between no/single morbidity and incident multimorbidity groups using Chi-square test for proportions and *t*-test for means. This was followed by bivariate logistic regression analysis to identify the variables significantly associated with incident multimorbidity. The variables found significant in the bivariate analysis at the p<0.25 level [32] were entered into multivariate analysis in different combinations to check their controlled/mediated effect on the outcome. The cases with baseline multimorbidity were eliminated from the logistic regression analysis to identify factors associated with incident cases of multimorbidity. The research team assessed the fit of the final model of independent long and short term determinants of incident multimorbidity using Hosmer-Lemeshow goodness-of-fit test and the area under the Receiver Operating Characteristic (ROC) curve.

The research team also calculated the adjusted relative risks for those determinants of incident multimorbidity, information on which was collected progressively [33]. The method of generalized linear regression with a log link, normal distribution, and robust variance estimator was used for adjusted relative risk estimation [34]. The analysis was conducted using SPSS-18 and STATA-10 statistical software. Missing values comprised 3.8% of cases included in logistic regression analysis and were treated as missing during the analysis.

## Results

The mean age of participants was 58.4 (SD 12.0) ranging from 39 to 90. Over two-third of them were women. The majority (69.1%) of the sample had secondary education and less than half (44.9%) were employed. The reported prevalence of multimorbidity in the total sample was 76.7%. In the older age group (65 years old and more) this rate was 86.7% while in the younger age group (39–64 years old) it was 72.5%. Among those with multimorbidity, hypertension and arthritis were the most commonly reported conditions (63.8% and 62.9%, respectively).

Table 1 provides the distribution of the selected baseline and current variables grouped in socioeconomic, health behavioral and psychosocial dimensions between those with baseline multimorbidity, incident multimorbidity and without multimorbidity. The group with incident multimorbidity was significantly older and less educated than the group without multimorbidity. Those with incident multimorbidity reported significantly lower employment rate and socio-economic status score, poorer perceived living standards during the first ten years following the earthquake, and lower affordability of healthcare services.

This group spent significantly less time on walking than those without multimorbidity. The information on health-related behavioral characteristics collected 22 years ago revealed statistically significant differences in body mass index (higher in the multimorbidity group) and the rates of regular participation in sport (lower in the multimorbidity group).

Variables from the baseline assessment related to the direct exposure to earthquake – earthquake-caused injuries, deaths among family members, and material loss, did not differ significantly between the groups with incident multimorbidity and without multimorbidity (Table 1). However, the life-time exposure to stress measured by the number of experienced stressful life events was significantly higher in the incident multimorbidity group. This group reported less social support and had lower memory and dignity scores compared to those without multimorbidity.

The findings of the bivariate logistic regression analysis confirmed the above-mentioned associations. The final multivariate logistic regression model identified six independent short and long term determinants of incident multimorbidity (Table 2). The number of stressful life events (both negative and positive) was strongly associated with multimorbidity. Each additional stressful event experienced by a respondent was associated with 15% higher chance of incident multimorbidity. This variable mediated the effect of age on multimorbidity, making age insignificant in the final model. Unlike current SES score, perceived living standards during the first ten years following the earthquake were also strongly related to incident multimorbidity in the final model. Rating family’s living standards as poor during the post-earthquake decade was related to 68% higher chance of incident multimorbidity.

**Table 2:** Logistic regression model on predictors of incident multimorbidity among a cohort of the 1988 Armenian earthquake survivors (N = 600, valid N = 577)

**Characteristics**	**OR**	**95% CI**	***P *value**
Current good social support	0.44	0.28–0.70	0.000
Number of stressful life events	1.15	1.07–1.25	0.000
Current low affordability of healthcare	2.36	1.56–3.58	0.000
Current university/higher education	0.56	0.36–0.87	0.010
Perceived poor living standards during post-earthquake 10 years	1.68	1.10–2.57	0.017
Baseline body mass index	1.06	1.01–1.11	0.026
*Model’s fit statistics:*	*Hosmer & Lemeshow goodness of fit test, p=0.803*		
	*Area under the ROC curve=0.752*		

*N* number of cases.

*OR* odds ratio.

*CI* confidence interval.

Three variables reflecting the current situation remained in the final model – perceived affordability of healthcare, social support, and education (Table 2). Perceived low affordability of healthcare was associated with 2.36 times higher chance of reporting incident multimorbidity. Good social support reduced the chance of incident multimorbidity over two times. University or higher education was associated with 44% lower chance of incident multimorbidity.

Of the behavioral variables obtained over two decades ago, BMI remained in the final model of predictors of incident multimorbidity. When controlling for all other significant variables, each one-point increase in BMI increased by 6% the chance of developing multimorbidity over this 22-year period. The final model achieved good fit indices – the p-value of 0.803 for Hosmer & Lemeshow goodness of fit test and the area of 0.752 under the ROC curve (Table 2).

Using the advantage the cohort study design provides, we calculated adjusted relative risks for the variables reflecting baseline characteristics of the participants – BMI at baseline, perceived living standards during the post-earthquake decade, and the number of stressful life events (Table 3). The findings were consistent with the results of multivariate logistic regression analysis – all three variables remained significant predictors of incident multimorbidity. When controlling for all other significant variables, the risk of developing multimorbidity was 12% higher for those with perceived poor living standards during the post-earthquake decade as compared to those with perceived average/good living standards during that period. On average, the risk of developing multimorbidity increased independently by 1% for each additional unit of the baseline BMI (kg/m2) and by 3% for each additional stressful life event over a respondent’s lifetime.

**Table 3:** Computed relative risks of developing incident multimorbidity for those with selected baseline characteristics among a cohort of the 1988 Armenian earthquake survivors (valid N = 577)*

**Characteristics**	RR^**^	**95% CI**	***P *value**
Baseline body mass index	1.01	1.00–1.02	0.006
Perceived poor living standards during post-earthquake 10 years	1.12	1.03–1.22	0.008
Number of stressful life events	1.03	1.02–1.04	0.000

*N* number of cases.

*CI* confidence interval.

^*^Generalized linear model, with a log link, normal distribution, and robust variance estimator.

^**^RR relative risk, adjusted also for current low affordability of healthcare, current good social support, and current education.

## Discussion

This study investigated the determinants of incident multimorbidity in the cohort of the 1988 Armenian earthquake survivors. The study design provided a unique opportunity to identify short and long term determinants of incident multimorbidity in this disadvantaged population group. Indeed, the information on some long term determinants of incident multimorbidity developed during the period 1990–2012 was collected prospectively. The six independent predictors of incident multimorbidity identified through this study included both current characteristics (social support, affordability of healthcare) and previous experiences reported retrospectively (number of stressful life events and poor living standards during the first post-earthquake decade) or prospectively in 1990 (BMI). Almost all these determinants were markers of social inequities or were closely related to inequities.

The prevalence of multimorbidity in our sample (76.7%) was closer to the higher end of estimates reported by other studies among similar age groups [7,26,35,36]. We believe that the high prevalence observed in this study could be attributable to continuous stress exposure of this population exacerbated by extremely high rates of poverty (more than 45% of the population) in the earthquake zone [37].

Although the majority of multimorbidity studies found that older age is associated with a higher risk of multimorbidity [3,6,19,21], the strong significant relationship between age and multimorbidity in our sample was largely mediated by the number of stressful life events, suggesting that experiencing stressful events during the lifespan might be a more important factor for incident multimorbidity than the lifespan itself. However, the stressful life events that our participants have lived through were more severe than that usually observed in the general population. Various studies have examined the role of stressful life events on morbidity, and the adverse effect of such events on mental and physical health is well established in the literature [38-40]. Nonetheless, we identified only one study that has examined the relation between life events and multimorbidity and found no independent association [41]. To our knowledge, the current study was the first to find that the number of stressful life events was associated with higher risk of multimorbidity.

The majority of studies investigating the association between BMI and multimorbidity were cross-sectional [19,41]; this study explored the long-term association between baseline BMI and incident multimorbidity. Lack of BMI measures from 2012 limited us in investigating the association between incident multimorbidity and different scenarios of changes in BMI over time. Nonetheless, this study suggests that BMI is an independent long-term predictor of incident multimorbidity with higher BMI values contributing to multimorbidity two decades later - a finding that deserves attention and further investigation.

Studies have reported that lower education is associated with higher rates of multimorbidity [3,21,26]. Our findings are consistent with this indicating less multimorbidity in those with higher education. Education is a well-established marker of social inequities in health. Its effect on multimorbidity might be due to both higher socio-economic status and healthier behaviors of individuals with higher education compared to those with lower education [42].

The positive impact of social support on health is highlighted in various studies. The significant association between concurrent social support and multimorbidity in our sample was also in line with Van den Akker et. al findings on the association of social network and multimorbidity [27]. Although the cross-sectional nature of the data on this variable does not provide the opportunity to check the direction of such association, it still indicates the beneficial effect of well-developed social networks on population’s health status.

Material conditions are among factors explaining the major role of social inequities in health [43] and multimorbidity [19,44]. In our sample those reporting lower living standards during the 10 years following the earthquake (1989–1999) had higher odds of suffering from multimorbidity in 2012. Interestingly, the current socioeconomic status score was not associated with incident multimorbidity in the final model. This association was largely mediated by the perceived living standards during the decade following the earthquake, suggesting an existing lag time between poor socioeconomic conditions and their impact on multimorbidity outcome.

In agreement with the identified adverse relation between socio-economic status and multimorbidity, we revealed that those reporting poor affordability of health care were at higher risk of having incident multimorbidity. This finding is consistent with an earlier study in Armenia, which identified low affordability of healthcare to be an independent predictor of self-rated poor health among women [45]. When we consider the higher need for utilizing health care services among those suffering from multiple health conditions, the low affordability of these services among them becomes an urgent and essential issue to address.

There were a number of limitations in the current study. The data we used were all self-reported, which can be subject to reporting bias. Some of our data were cross-sectional limiting us to draw conclusions on the direction of the causality of the associations. Our study population was a cohort of earthquake survivors who lived in extremely difficult conditions during the last two decades. Therefore, caution is needed when generalizing the findings of this study among other populations.

As the majority of the independent determinants of incident multimorbidity identified through this study were related to social inequities, developing and implementing strategies targeting social inequities would be important for this population as well as for other disadvantaged population groups in other countries. This study also highlights the importance of promotion of healthy lifestyle and weight control starting from younger ages. Building well-functioning social networks could further reduce multimorbidity in the areas affected by natural and man-made disasters. The findings of this study could be applicable for improving health outcomes in other countries with social, economic and wealth distribution profiles comparable to that in Armenia.

## Competing interests

VP is an associate editor (unpaid) at the International Journal for Equity in Health.

## Authors’ contributions

All authors participated in conceptualizing the study and designing the survey. AD performed the analysis, AD and VK drafted the manuscript. HA and VP substantially contributed to the interpretation of data and critically revised the manuscript. All authors read and approved the final manuscript.
